# Health and social concerns about living in three communities affected by per- and polyfluoroalkyl substances (PFAS): A qualitative study in Australia

**DOI:** 10.1371/journal.pone.0245141

**Published:** 2021-01-14

**Authors:** Cathy Banwell, Tambri Housen, Kayla Smurthwaite, Susan Trevenar, Liz Walker, Katherine Todd, May Rosas, Martyn Kirk

**Affiliations:** 1 National Centre for Epidemiology and Population Health, Research School of Population Health, The Australian National University, Canberra, ACT, Australia; 2 Ngaigu-Mulu Aboriginal Corporation, Katherine, NT, Australia; East Carolina University, UNITED STATES

## Abstract

**Background:**

Exposure to per- and polyfluoroalkyl substances (PFAS) is a public health issue globally. In Australia high concentrations of PFAS have been found in environments close to sites where Aqueous Film Forming Foams (AFFF) were historically used for firefighting activities. This has resulted in significant community concern about the potential long-term health effects of these chemicals.

**Objective:**

We describe residents’ perceptions and experiences of PFAS in three regional Australian towns where exposure has occurred.

**Methods:**

We conducted focus groups to generate free-flowing open discussion on PFAS in three affected communities, including some with significant numbers of First Nations Peoples. We recruited participants using a range of media outlets and postal services. Focus group transcripts were analysed thematically to identify major shared concerns using Atlas Ti.

**Results:**

One hundred and eighty residents attended fifteen focus groups that were conducted in the three communities. They included 69 First Nations People living in three communities near the town of Katherine in the Northern Territory. Study participants were concerned about potential physical health effects of exposure to PFAS, such as cancer clusters, unexplained deaths, potential exacerbation of existing health conditions, and the future health of their children. They expressed feelings of stress and anxiety about living with uncertainty related to the possible health and the socio-economic impacts of PFAS contamination in their communities.

**Conclusion:**

While research has concentrated on the physical health effects of PFAS, more attention needs to be given to the immediate psychosocial impacts of living in an affected community.

## Introduction

Human exposure to per- and polyfluoroalkyl substances (PFAS) is an issue of global public health importance. PFAS are highly inert, synthetic substances produced since the 1950s for a wide range of consumer products and industrial applications [1]. The extensive use of PFAS led to substantial environmental distribution of the chemicals, resulting in uptake by animals and humans [2, 3]. PFAS has been detected in human blood serum samples from the general population. However, consuming food and drinking water from PFAS contaminated environments may result in people having higher blood serum concentrations of PFAS than the general population [4].

Environmental contamination by PFAS has generated significant community concern about the potential long-term health effects. Research has investigated exposure pathways and epidemiological associations between PFAS and a range of human health outcomes, such as metabolic, reproductive, neurodevelopmental and immunological effects [5]. Studies of populations with elevated exposure levels have reported an increased risk of dyslipidaemia with increasing blood serum PFAS concentrations, in particular perfluorooctanoic acid (PFOA) and perfluorooctane sulfonic acid (PFOS) [6–9]. While this finding is supported by several epidemiological studies of the general population [10–12], the biological pathway of this effect is unclear in humans [13, 14]. Recent systematic reviews have also reported additional health outcomes associated with exposure to PFAS, including: decreased renal function and increased risk of renal cancer; and inverse immune outcomes in children, such as reduced vaccine-derived immunity against specific vaccine preventable infections [15–17]. However, uncertainty remains for many other health effects resulting from exposure to this class of chemicals.

It is now widely accepted that proximity to environmental contaminations may affect residents’ psychosocial health [18]. Studies have shown that human-made, slowly-evolving environmental disasters [19], such as mountain top mining [20] or oil spills [21], have negative social and psychological impacts on communities [22, 23], particularly those that are disadvantaged [24, 25]. As environmental disasters unfold and persist they can undermine the psychological resilience of communities [26, 27] resulting in trauma, stress, and loss of trust [28], and social capital [29]. Communities may become divided, struggle for a collective response, and experience economic vulnerability. For example, the BP deepwater horizon oil spill is cited as an example of institutional recreancy linked with heightened community distrust, and the need for better risk communication and resident involvement [30].

### The Australian PFAS experience

PFAS were first introduced to Australia in the 1950s and used for firefighting activities on Defence Force bases from the 1970s. Aqueous Film Forming Foams (AFFF) containing PFOA and PFOS were phased out of use by the Defence Force in 2004 [31]. Concern about PFAS has grown across Australia since then, stimulated in part by class action suites and by visits from Erin Brockovich who labelled the Australian Government’s response at the time as “inadequate” [32].

**Fig 1:**
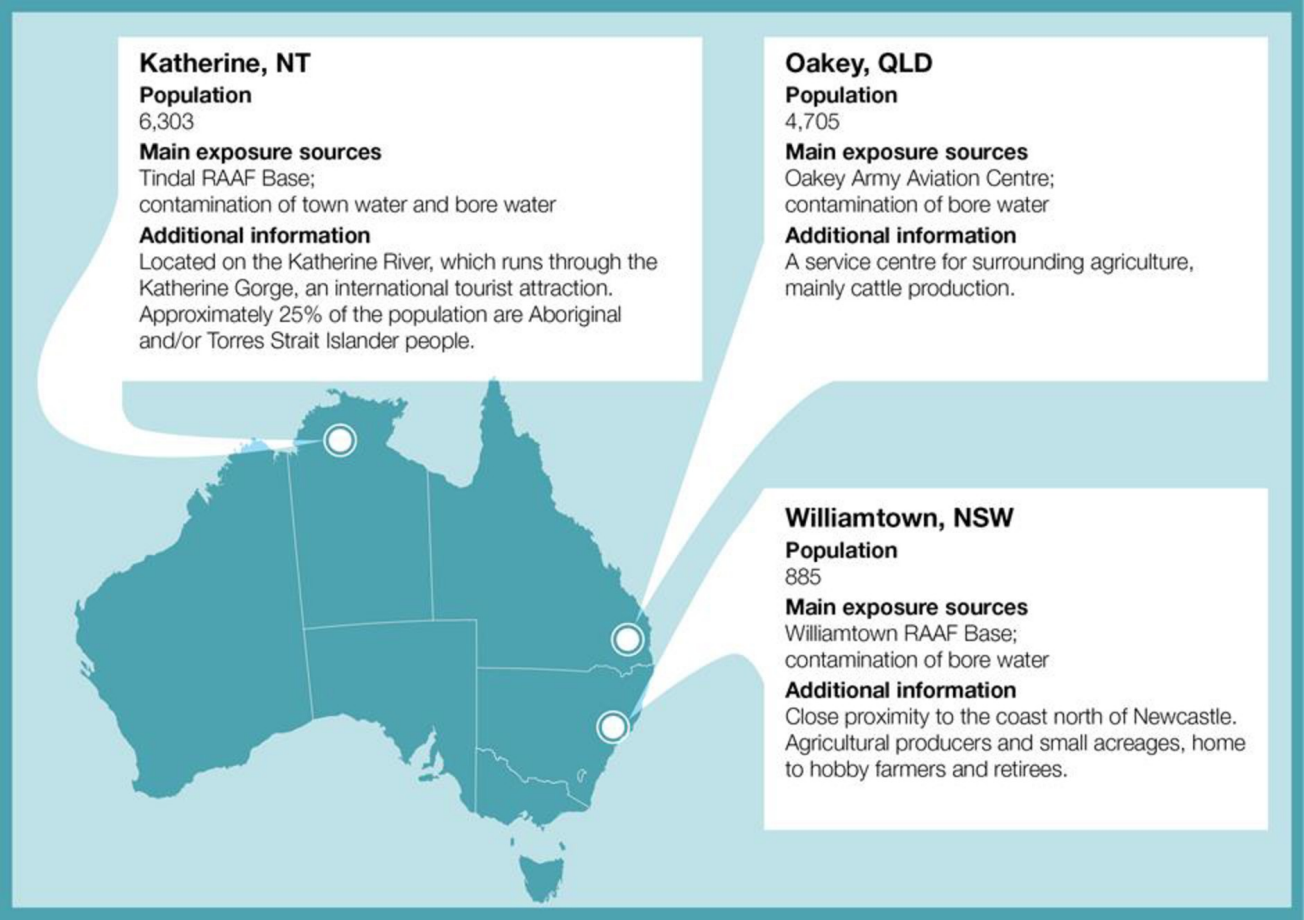
Characteristics of the PFAS affected communities of Williamtown, Oakey and Katherine. Population data sourced from QuickStats, Australian Census, 2016 (Australian Bureau of Statistics 2016).

While PFAS has been widely used across Australia for domestic and industrial purposes, three regional towns have been singled out for government action due to heavy use of PFAS on nearby Australian Defence Force bases. They are: Williamtown in New South Wales, Oakey in Queensland and Katherine in the Northern Territory (Fig 1). All three communities are situated on flood-prone areas located within a 10km radius of the bases where PFAS were used for firefighting. Residents and workers of these communities have been potentially exposed to PFAS through the consumption of contaminated ground (bore) water and town water, and from local produce, among other exposure pathways. The Australian Government Department of Health established a PFAS coordination unit to facilitate health related work in Oakey and Williamtown and in late 2017 extended it to Katherine. Part of this work was to measure community serum PFAS levels and commission the current PFAS Health Study to investigate exposure to and the health effects of PFAS and to assist policy makers’ response to PFAS contamination issues.

Thus far, little scholarly attention has been paid to the psychosocial health effects of living in PFAS-contaminated areas due in part to the relatively recent acknowledgement of its potential environmental impacts by the Australian government. In this paper, we analyse the experiences of people living, working or owning property in a PFAS affected community. Our aim was to understand residents’ and workers’ perceptions of health and other PFAS exposure risks in these three regional Australian towns where community exposure to PFAS has been measured. This exploratory qualitative study is part of the larger study, currently in progress.

## Materials and methods

This focus group study was the first phase in a sequential mixed-methods study—The PFAS Health Study (pfas.anu.edu.au) that includes a cross-sectional survey to investigate socio-demographic and other risk factors associated with high serum PFAS, health problems and psychological distress, a study comparing blood serum levels of residents and non-residents, and a data linkage study of PFAS candidate diseases among people living in affected areas and the general population. As focus group discussions are inductive and exploratory they are well suited to collecting data to inform quantitative research [33]. Group-interviews were preferred over in-depth interviews as they facilitate discussions of community events that generate shared public knowledge, underlying attitudes, and perceptions and opinions. This method suits exploration of a range of views on community topics [34], which may reveal concerns and issues generated by group interactions and have been used in similar research elsewhere [30]. Interpretivist group discussions such as these, focus on participants’ meaning of reality [35]. Rather than aiming for statistical representativeness, we intended to capture a range of community views and experiences related to PFAS contamination. In addition to providing early insight into residents’ perceptions and experiences, community discourse and concerns captured during the focus groups will inform the development of the survey and contribute to synthesis of qualitative and quantitative findings [33].

### Recruitment and data collection

We conducted twelve focus group discussions with community residents and workers in Williamtown, Oakey and Katherine between January and August 2018 (Table 1). A further three focus groups of 69 people were held specifically in communities with First Nations People in Katherine. In total, 180 people attended 15 focus group discussions. We recruited community members at each location through advertisements using traditional and social media, including websites and community reference groups. Residents and workers who were interested in participating in the focus groups registered with the study team. Participants were sent a detailed participant information sheet about the nature of the discussions. To recruit participants from three First Nations communities in Katherine, an Aboriginal Elder visited each community to explain to community members the purpose and content of the focus groups, and agree when the team could visit their community. The Elder was consulted earlier about how to introduce and conduct the study and she facilitated the focus groups.

**Table 1:** Composition of focus group discussions, PFAS health study, Australia, February-August 2018.

	Oakey 4 Focus Groups (*n* = 36)	Williamtown 4 Focus Groups (*n* = 46)	Katherine 4 Focus Groups (*n* = 29)
Male	58%	46%	34%
>50 years old	75%	65%	66%
Education			
Incomplete secondary	22%	6%	10%
Secondary	30%	41%	10%
Certificate/Diploma	25%	37%	34%
Bachelor or above	22%	15%	45%
Married or co-habiting	83%	74%	55%
Employment			
Casual employment	3%	4%	11%
Part-time employment	6%	4%	0%
Full-time employment	50%	39%	43%
Retired	35%	46%	39%
Unemployed	6%	7%	7%
Children living at home	36%	65%	34%
Own home	92%	80%	72%
Lived in area 10 or more years	72%	65%	89%

(Note table does not include participants from First Nations focus groups.)

A research team led by an experienced qualitative researcher conducted group discussion sessions during the day and at night in a convenient central location to enable a range of participants to attend. In each town, we conducted one separate focus group discussion with people connected to the local Australian Government Department of Defence site, including workers and immediate family members. Some residents indicated that they joined focus groups with people whom they knew shared similar views so that potential discord within groups was reduced. The facilitator and team made opportunities for less vocal groups members to speak.

All study participants provided informed consent and completed a brief socio-demographic questionnaire. The team provided information on local counselling services at the start of each discussion and participants were able to leave the discussions at any time. Participants were asked to adopt a pseudonym to reduce the risk of their real names being inadvertently used in the discussions. Maintaining confidentiality and privacy for all was important and participants were asked to refrain from discussing focus group discussion contents with others in the community.

An Elder facilitated discussions with Katherine First Nations Peoples on their community lands over a two-day period. Participants were not asked to complete the socio-demographic questionnaire as the discussions were adapted to be culturally appropriate. They were informal and included large numbers of people who moved about. Most participants were women, often with children, with men sitting separately on the margins. All the group discussions were held in central outdoor facilities. The communities varied somewhat in size and distance from Katherine and represented the main First Nation communities in the area affected by PFAS contamination.

We used an interview guide to generate discussion covering the following broad areas: health; risk perception and understanding of PFAS exposure; emotional responses; stigma; practical issues; connection to local land and water sources; and perceptions of the media’s reporting of PFAS. Each discussion lasted between one and two hours. All focus group discussions were audio-recorded with consent and transcribed. The focus group discussions did not obtain information about the results of blood tests carried out by the Departments of Health or Defence, land or water contamination levels or actual government remediation or communication strategies. Our aim was to understand people’s perceptions of these experiences.

### Data analysis

After each focus group, study team members discussed and made joint field-notes of the mood of the group and significant statements made by participants. We used thematic analysis to provide “a rich and detailed, yet complex account of data” [36]. After familiarisation with the content of the transcripts, we constructed a mixed inductive and deductive coding matrix [36] before manually applying code words and phrases to the texts. These were later entered into a qualitative software package (Atlas ti version 8) to assist in data management. We grouped coded segments of text into broader level themes that loosely aligned with the aims of the study although we monitored for unexpected findings.

### Ethics

This project was approved by the Australian National University Human Research Ethics Committee (Protocol 2017/816, 2018/151), the Departments of Defence and Veterans Affairs Human Research Ethics Committee (Protocol 024–17, 055–018) and the Northern Territory Department of Health and Menzies School of Health Research Human Research Ethics Committee (Protocol 2018–3121).

## Results

A total of 111 people participated in 12 focus groups in the three communities (Table 1). On average, participants were over 50 years, and were either retired or working full-time. Most owned their own homes and had lived in the area for long periods of time. On average, focus groups participants differed from the general population by being older, better educated and less likely to be employed full time compared to the Australian Bureau of Statistics 2016 census data. Katherine participants included slightly more women, and more people with tertiary education. Focus group members were not recruited to be statistically representative. They appeared to be people who had a long-term commitment to the area in which they lived.

This paper is organised to reflect five major inductive and deductive inter-related themes arising from our analysis: challenging exposures, questioning health, financial uncertainty, eroding trust, and finally stigma and disunity. First Nations people shared elements of overarching themes such as concern for their own and their children’s health, but their fundamental relationship to land and property was so different that their results are presented separately.

### Challenging exposures

In all three communities, participants told us about, and challenged, the way in which PFAS levels were measured in water and soil by consultants commissioned by the Department of Defence. This testing had occurred before focus groups were held. Participants disputed the boundaries of PFAS contaminated areas as arbitrary, particularly when someone living on one side of a street was affected while neighbours on the other side were not. Representatives from the Commonwealth Departments of Defence and Health then held town hall meetings in the communities to inform the communities about sources of PFAS contamination. Participants understood that it was the PFAS contamination originating from Defence Force bases in each affected community which had then brought them into contact with the chemicals through bore and surface water on their properties, consuming local produce and, in Katherine, the use and consumption of contaminated town water. Older residents of Oakey and Williamtown also told stories about years past before the risks were known, when firefighters sprayed foam at community Christmas events, presumably to replicate snow, for children to play in. It was clear that participants perceived the movement of PFAS contaminated water through their environment as dynamic and contested. The boundaries of affected areas have been remeasured as they have shifted over time, creating confusion and uncertainty. Questions remained about the adequacy of treatment of drinking water in some communities.

Participants reported being advised by government representatives not to eat fresh produce grown on their properties using this contaminated groundwater, including eggs from their chickens and to minimize consumption of their livestock and products from livestock. Rural residents who were transferred from bore water to town water to reduce exposure reported that this water was too expensive for agricultural purposes and it increased operating costs. All three affected communities had experienced severe floods, including several in recent years. Some participants attributed PFAS polluted topsoil, even on land outside areas of investigation, to these events which in turn added to their worries about contaminated produce and livestock. Some rural properties were remediated with new top soil or were given rainwater storage tanks to replace bore water.

Discrepancies between the scientific evidence and personal experience intensified participants’ confusion and uncertainty. Participants said that there were “a lot of unknowns”, and a “lot of misinformation” about PFAS exposure. During discussions, they generated the sense that almost every aspect of PFAS contamination and exposure was unclear, leaving them doubtful about how to respond or move forward. Their difficulty in determining their exposure to PFAS contributed to their uncertainty about the potential health effects.

The differing published research findings also contributed to community concerns. Some participants had located large numbers of studies online containing an array of findings that could support their view. This contributed to the distrust of government agencies’ information and advice, which tended to show that health risks were lower than reported in the international studies participants had found on the internet.

### Questioning health

Participants sought ways to assess their physical health risks based on a combination of scientific reports, local knowledge and everyday experiences. A few were reassured because local wildlife and livestock living in contaminated areas were apparently healthy. One person questioned expert advice to not consume home-grown agricultural produce saying that local “animals would know what is good water and what is not”. In contrast, some livestock producers observed a significant reduction in animals’ fertility and an increase in stillbirths and other health problems, which they postulated was due to PFAS exposure.

Many participants expressed fears for their future health and even more so for their children’s health. They were concerned that they had subjected their children to an increased risk of developing diseases, particularly cancer, as well as increasing their own risk of cancers. They were convinced that what they understood as “cancer clusters” in their area were due to PFAS exposure. One person noted that “there were 51 with cancer when we went to the last meeting in one community”. A very few people, like this participant, however expressed uncertainty about this.

“Is there a spike in bowel cancer in the area because people have got it in their family? Prostate cancer, it’s an aging disease, is that why we’ve got so much of it around, we’re living longer, or is it something to do with the PFAS exposure? Nobody knows”.

Apart from cancers, participants generally did not attribute specific health conditions to PFAS. Several wondered whether an existing health condition, usually breast or prostate cancer or in one case a chronic degenerative disease might have been either caused or exacerbated by exposure to PFAS. Participants who worked on the Defence Force bases speculated about the risks of long-term health outcomes. They and others like them had been exposed to high levels of PFAS in the past, some directly through contact with firefighting foams. Several recalled themselves or their children experiencing immediate skin symptoms following direct exposure to the foams or bore water on the bases, which they described as “itchiness”, “hives” and “skin crawling”. However, as veterans of the Australian Defence Forces they were eligible for government supported health care.

In Katherine, participants were concerned about swimming in, and drinking water from, the Katherine River which was a major recreation site for the area. They also expressed concern for the health of ‘Country’ in the context of their attachment to the land and environment in which they live.

Participants, such as this one, linked psychosocial and physical health.

“My main concern is the long term health of the people who are well above the average PFAS content in their blood. And the second main concern is the mental health of the people … that have been subjected to extreme pressure. Not only of the blood tests, but also of their property values and their lifestyle”.

Although participants usually cited their physical health as their primary concern, their discussions often focused on their emotional responses to their current situation. A participant observed that community members “talk about stress of health and mental stress” and another group member from the same town thought that their community was brought together by “the anger and negativity”, going on to say:

“That’s the mental side of it. Is that you don’t know, you get sick and you don’t know whether to [attribute] it to the pollution here … and there’s that psychological side. That you sort of—is what I’ve got caused by this or is it something else?”

While participants acknowledged that it was “very difficult to prove” the link between PFAS and physical health problems, they said “it’s still there in your mind”. They observed that local residents were “stressing, [they] are angry … about their house”. One person commented that contradictions in their situation exacerbated their poor mental state.

“This block of land has been my home, it’s where I brought up my kids, its everything to me and yet at the same time it’s a contaminated piece of land and you’re holding these two things in your hand, and the confusion of it, these two opposing truths, it drives you nuts”.

Anger and guilt was heightened by fear that they had potentially exposed their children to chemicals, often since they were young. As one person noted: “I don’t have the right to contaminate my grandkids”. Another participant explained:

“We’ve got two teenage kids. And [its] the [health] unknown of [that] they’ve been eating our fruit and vegetables that we’ve been growing until recently. And we were told that it was safe to do so”.

Participants were monitoring, obtaining and comparing information about PFAS; they felt that this time could have been better spent on activities like generating income or on leisure. They primarily associated their stress, anxiety and anger with uncertainty over long-term health prospects, and with disruptions to their social and economic situation.

Some participants added that the need to respond to potential physical health problems aggravated their poorer mental health. For example, one person observed that getting pre-test counselling for blood testing had resulted in them feeling in “worse shape”. However, others acknowledged that it was almost impossible to know whether future health problems could be attributed to PFAS exposure.

### Financial uncertainty

Participants observed that there had been a major decline in property and house prices due to PFAS contamination. One person described the low relative value of a small semi-rural property equivalent to eight hectares:

“20 acres, $100,000 four bedroom house. I don’t know anywhere else in Australia that you’d get something like that, maybe [the other PFAS affected sites]”.

People living in affected communities stated they were unable to sell or improve their properties because banks had stopped lending money on homes in the ‘contamination area’. Several said that they could not get their properties even valued. The banks applied the same approach to non-residential business properties, suggesting that lending policies were based primarily on a financial rather than health risk assessment. They were unwilling to maintain and improve properties if they could not recoup the costs but were distressed to see their properties deteriorating.

Several participants had bought properties which they planned to sell later to finance their retirement and aged care. They contemplated loss of financial independence simultaneously with the burdens of ill-health and aging. An older person explained:

“We’re caught. If I spend my super [retirement savings] and buy another house I won’t have any money to live. Certainly, I’m not happy. I was happy here for a long time, or reasonably happy but since this started, I don’t want to live here anymore”.

Furthermore, they did not want to pass on their contaminated properties to children because of health and financial risks as this person observed. “I’ve got a property there that my kids wouldn’t be able to sell and they don’t want it, they sure don’t want it”. Other participants wanted to move elsewhere but were unable to sell their properties causing them to say they feel “stuck”—a term which was widely used in all the focus groups.

Attempts to minimize exposure to PFAS imposed additional financial burdens. Participants reported buying bottled water despite assurances that the free town water was safe. People living on rural land purchased town water for agricultural purposes rather than using free bore water. Some were forced to purchase additional food on the advice not to consume products from their properties due to PFAS.

“I wanted [Defence] to shut my bore down and compensate for my cost of the bore and then to pay my excess water and use town water. And they said no but yet they told me don’t drink the water and don’t put it on your veggie patch”.

Many did not have high disposable incomes or had chosen a sustainable and self-sufficient lifestyle, while others were rural producers who raised livestock or grew fresh produce that they either sold commercially or consumed themselves. Commercial producers were concerned about reputational damage and loss of business income. They felt uncomfortable selling livestock grown in affected areas, despite receiving advice that it was safe for commercial sale. Some stopped raising animals, euthanized livestock, and replaced contaminated soil because of financial losses. One person explained the difficulty in leaving their property which had economic and emotional value, and representing deep family connections to identity and place. “We’ve been there 30 years and you can’t walk away and where do you live?” Another participant added:

“We built the place. We dug it from the ground up and brought our kids up there and there’s a lot of emotional attachment to it. It is not just financial”.

### Eroding trust

Participants expressed high levels of suspicion and mistrust resulting from PFAS contamination. They felt that they had been deliberately misinformed or dismissed by government officials from the Departments of Health and Defence during community meetings because questions were avoided or left unanswered, and the information they received was insufficient or contradictory. One person told us they became more angry after attending a meeting, because of the way the meeting was conducted and community members were treated. Participants were particularly suspicious of the Australian Government Department of Defence and mentioned conflict of interest because members of the affected bases had been involved in managing the response to PFAS. Others differentiated between the Defence Force as a Federal Government institution, and local Defence Force personnel, whom they considered to be absolved of responsibility for the contamination. However, several Defence Force personnel thought they were viewed negatively by community members.

There was a level of suspicion of other stakeholder groups, such as real estate agents and lawyers. Real estate agents were reported to sell properties without disclosing the PFAS problem to buyers while an ongoing lawsuit exacerbated tensions between community groups. In one community, people thought that contamination boundary lines were drawn to exclude “big businesses that are worth millions”. Some participants who had made submissions or had followed the proceedings closely of an Australian Government Senate Enquiry into PFAS observed that none of the recommendations had been adopted [37, 38].

Community members had differing views on the role of the media when reporting health effects and the scale of contamination. Generally, the media was seen as a useful tool for attracting attention to their concerns and providing a mechanism to nudge government. Others thought the media was purely interested in using the PFAS story to sell content, irrespective of whether the story was true or damaging to the community. Some distrusted the media almost as much as government representatives, bureaucrats and other experts because their “scare-mongering” and “sensationalism” contributed to community stigma.

Affected communities were highly suspicious of information provided by government agencies associated with PFAS. This distrust affected the reception of public health research into PFAS. Some participants associated researchers in general with government and others felt that monies could be better spent on finding a solution, or that it would take too long to conduct the research which would allow the government to avoid responsibility.

Distrust was linked to a sense of injustice and unfairness. Participants described themselves as hard-working Australians who had paid taxes and contributed to their community. Some thought that the government was failing in its role to protect them as citizens. Several commented that if they were to contaminate their neighbors’ land they would be expected to pay, but because a government agency was the source of contamination there was no accountability; they felt that there were two sets of rules. Members of all three communities commented that their geographic distance from the center of Federal Government diminished their influence on the policy decisions and interventions regarding PFAS. A further reflection came from a participant who observed that the experts and bureaucrats who visited them:

“…[they] don’t have to live here you see, they can breeze in and breeze out, the same with the pollies [politicians]. I’ve seen dozens of them, they come in and promise you this and that and they’re gone, never see them again”.

Paradoxically, the Commonwealth Department of Health’s program of free voluntary blood testing may have increased community confusion and distrust. The program was established in response to community demands when the PFAS contamination became widely known. The bloods were tested at a national private laboratory and the results were fed back to community members through local medical staff. At the time of this research, some people had paid for their own blood tests, while others had participated in the free government program.

Discussion among focus group participants suggested that people who had been tested had varying experiences of the program. They were often unclear about the meaning of results, which were not well explained by local doctors, leading to further confusion, uncertainty and distress. Participants said they were not provided with baseline measures with which to compare their results. Participants found that PFAS levels in affected residents’ blood did not match with the measures of PFAS in environmental samples. For example, people said that produce from their property showed high PFAS levels, while their own blood tests were low or *vice versa*. Community members compared results and discussed discrepancies among themselves. A repeated concern was how to respond to the results of blood tests particularly if they showed high levels of PFAS. A great source of stress was high PFAS blood levels in children. Some participants chose not to get their children tested to avoid the anxiety of high PFAS levels. There was also confusion about whether the purpose of the tests was to determine individual or community PFAS contamination levels.

### Stigma and disunity

Segments of communities that were agitating for government assistance formed support and advocacy groups. Some joined a class action suit led by a commercial law firm which brought Erin Brockovich the famous environmental activist to the communities, to encourage people to sign up. Groups collaborated with each other, forming channels of communication between the three communities while deepening divisions within groups in their own community who wanted the issue ignored. Widespread media reporting across Australia of PFAS contamination of the three communities also contributed to disunity. Residents were concerned that their communities had become publicized for having non-potable water, which had a negative effect on tourism and agricultural businesses. Others encouraged press coverage to pressure the government into responding.

In all three affected communities, participants were asked how they would move forward from PFAS contamination. They envisaged a range of solutions consistent with the diversity of their views. Some wanted to move away from contaminated land but could not afford to because of the drop in property values, so they wanted financial support. Others wanted to remain where they currently lived if the health risk was removed. One person said, “I want them to decontaminate my property, to make it livable”. Yet others wanted to be confident about the health effects of PFAS so they could make an “informed” decision. They hoped that good research into the problem would provide this. Participants living in the town of Katherine were more likely than others to say that they did not want to move. Part of this was the expressed attachment to Country and to the Katherine River. Members of the general and First Nations communities resented being told not fish or swim in the river. Participants also wanted greater transparency in government’s response to PFAS and clearer information. They said that the government agencies should listen to them and treat them respectfully.

### First Nations perspectives

First Nations participants were concerned about the potential impact of PFAS on their own health, and particularly on the health of their children “and their future”. This concern or “worry” was exacerbated by high levels of existing illness in the communities, which left participants wondering if PFAS had contributed. One person observed that a “lot of people die … too young” and another said “there’s a lot of sick people in the community”. A woman questioned whether the recent death of a child was due to sickness from PFAS while another person said “when I water [plants], if it touches your skin, I’m thinking about you might get cancer the long way round [over time]”. Participants linked their health concerns about PFAS to the “health of Country” an expression which refers to an “interdependent relationship between Indigenous peoples and their ancestral lands and seas” [39].

There was little discussion of mental health concerns in these groups, except for general statements about being “worried”. Apart from these comments, participants generally did not link PFAS with specific health conditions. First Nations members’ perceptions of their poor health appeared to intensify their feelings of vulnerability to PFAS. However, PFAS seemed less immediate to them than their existing health concerns.

First Nations participants appeared uncertain about the impact of PFAS on their environment. Most people had heard or had seen signs saying that they should only eat one river fish a day because the river and the fish in it were contaminated. The advice to limit fish consumption had considerable cultural and economic significance. People said that they had previously fished frequently in the river and often consumed large quantities of fish of different types, turtles and their eggs, mussels, as well as crocodiles and their eggs. They talked about PFAS as though it was a disease that people could catch from contact with river water or river foods–if “we eat the fish that’s infected, do you think that we’ll get infected too?” People were unaware that some types of fish were less contaminated with PFAS than others and were therefore safer to eat. They requested more information about the safety of tap water, organ meat from local animals, and river foods, such as eggs, shellfish and crocodiles.

Most First Nations participants did not want to move elsewhere because of PFAS—as one person said, “This is our home”. They were unsure about whether their land had been contaminated during previous floods, and whether it had been tested for PFAS. One person observed: “They damaged the Country, yeah, they poisoned the river”. Running through an often dry landscape, the river was central to many of their activities and recreation with a person commenting that: “the river is our life”. Someone wondered why “they say that you can go swimming and that, but don’t drink the water”. Nevertheless, people were unclear about the safety of swimming in the river particularly for children.

Although it was difficult to estimate, First Nations participants said that they spent more of their income than previously, or a “lot of money”, on supermarket food to replace bush foods and fish. Many now bought bottled water. Participants also reported that the bush foods bought in the shops “like kangaroo, crocodile, whatever doesn’t taste the same like the bush one” and that they had replaced wild meat and fish with farmed red meat, suggesting that the PFAS contamination may contribute to a move away from traditional diets.

First Nations participants said that they did not attend community information sessions run by government representatives because they were located in places in which they did not feel comfortable. Overall, they did not appear to have received the same level of detailed and specific knowledge that many townspeople had obtained. Others mentioned that they heard about it from the media or from other people. They were upset that the dangers of PFAS had been known about long before they were informed. Referring to the importance of guidance in managing Country from older custodians they asked:

“But why we weren’t told back then, you know, in the ‘80s, we weren’t told. And we had, our old people were still alive, and they didn’t speak and tell our Elders while they were still alive”.

From their accounts, it seems that little had been done to explicitly develop communication strategies for their communities.

## Discussion and conclusion

In this study, PFAS-affected communities in three rural communities in Australia reported concerns about health, financial impacts, distrust of government agencies, and stigmatisation of communities. They felt that the risk of PFAS had been downplayed by government agencies and that it had been difficult to draw attention to their plight. Participants across all three communities expressed broadly similar understandings and concerns about PFAS, related in part to dwelling in and around small towns that catered to agricultural production and in Katherine’s case a strong tourist industry and the nearby First Nations communities. Residents who were concerned about PFAS shared information and advice between the three locations which may have contributed to similar views expressed by residents across all three communities.

Within communities there were differences of opinion between those who wanted to publicise the problem in order to provoke a more supportive government response, while others preferred that it be downplayed so that tourism or local business wouldn’t be affected. Generally, professional and hobby farmers and domestic property owners were more concerned about economic and reputational damage from contamination than town-based and tourist businesses.

A somewhat different perspective was offered by some people who had worked on the Defence Force bases. Despite their fears of heavy PFAS exposure in the past, they were more likely to contextualise their health concerns in light of their workplace exposure to other toxicants, and hazard reduction training. As most of them no longer lived in contaminated areas they were less affected by the social and financial implications and their healthcare was provided through the Defence Force. Those who remained in contaminated areas shared similar concerns with other local residents.

The major differences between the sites related to the experiences of Katherine’s First Nations communities who had few economic assets and did not own properties individually. They focussed on their connection to Country, the effects on land and waterways, and loss of access to indigenous foods and rather than diminishing property values. In this regard their concerns were similar to the Mohawk people experiences of PFAS contamination in North America [40]. The health and environmental complexities of PFAS are driven by interactive, dynamic elements that challenge experts and community members alike to explain and understand them. Qualitative approaches, such as this, help as they are considered “important to community environmental health research, as they give voice to individuals and community-based organizations and characterize the community in a full and complex fashion” [41]. Community members in our study were confronted by the pervasiveness of the impact of PFAS on their everyday lives. The plethora of contradictory evidence on the internet and elsewhere resulted in confusion and stress as people tried to navigate information to inform themselves about potential health risks. Overwhelming concerns led them to seek clarity and certainty, sometimes by attempting to identify an individual component that they thought could produce an easy, quick solution. These feelings were exacerbated by the slowly unfolding nature of PFAS contamination, delays in authorities’ responses, uncertain health effects, perceived lack of transparency and poor risk communication.

Generally, communities experience greater negative emotional effects in response to human-made disasters [42]. These contamination events also differed from many of the US major industry-generated contamination events because they occurred though government-related activities. Our study suggests a future research topic would be to investigate whether responses from affected communities was influenced by which organisations generated the contamination. Community members acknowledged that the Australian government had responded but criticised it for downplaying the risks and for being insensitive, slow and unhelpful. The blood testing program was prompted by community demands but generated considerable anxiety, particularly for people with a high blood concentration of PFAS because the results were difficult to interpret or lead to a course of action. It seems that research recommendations to develop specific protocols to inform and engage communities in research [43] were not well employed, exacerbating negative views of government. Overall, some participants’ sense of injustice was heightened because they believed that their government should have protected them more quickly and effectively.

First Nations participants felt that their unique dietary and cultural practices were not factored into risk assessments and communication. The location of community consultations and information meetings excluded First Nations People and contributed to their lower awareness of PFAS and how to minimize risk of exposure. It appears that there had been minimal attempts by government agencies to develop communications designed specifically for these communities. The relationship with Country is vital to Australian First Nations People, holding spiritual, cultural, traditional significance as well as being a source of livelihood. In this regard, they share loss of traditional foods from culturally significant sites such as rivers with other First Nations People [40]. In keeping with other research findings, greater effort was needed to ensure Indigenous populations are fully informed and consulted with. In discussions they requested information on the impact of PFAS on traditional practices such as hunting, eating bush foods and fishing [44–47].

The social and economic impact of PFAS contamination such as land devaluation, difficulty selling contaminated properties, and loss of financial security was discussed in focus groups. Future research could provide useful quantitative economic analysis. Participants had been forced to alter retirement plans, travel, holidays and financial planning, and felt deprived of being able to leave a legacy for their children. Food producers could not to eat their own produce even though they could sell it on the market.

This study, with others, promotes an understanding that including people’s experiences may usefully inform more effective risk communication [35, 48, 49] that is transparent, clear and empathetic [50]. The perceived lack of transparency from visiting government officials fuelled community members’ distrust. Research has found that engaging communities early in environmental contamination events and providing a safe space to express concerns [51] improves communication and trust. Community involvement in the co-design of responses has been shown to provide a greater sense of control and reduce negative mental health effects [52]. For First Nations communities, it is vital to recognise their close connection to land, the importance of physical, socio-economic and cultural well-being, and to strengthen their capacity to “identify, understand and control impacts associated with development” [53].

PFAS contamination joins the list of environmental disasters that are difficult to detect, evolve slowly, involve human causality and are persistent, thus creating existential uncertainty [19]. Often the government or official response to these events is mainly scientific and excludes subjective experiences even though risk uncertainty, mistrust, confusion, and outrage are common community responses [54]. Accordingly, these complex events uneasily span the scientific world of risk assessment and lived experience [55] contributing to competing explanations and understanding. PFAS contamination, like other environmental contaminations, mainly attracts a scientific response [56], although it is characterized by “ambiguity and conflict regarding [its] nature and impact” [19] which contributes to individual and community level stress. In these situations, it is also difficult to define who are the victims and what are their needs [19]. It has been observed that when faced with contamination events over time, social and familial relationships may fracture, community tensions rise, and social capital declines as residents leave [26, 27]. The Australian PFAS affected communities displayed some elements of declining community capital, such as dissension, despite efforts to build and maintain social bonds in the face of adversity.

Focus groups discussions are designed to capture a range of shared community experiences and views through group interactions rather than sensitive individual perceptions and experiences. The self-selection of participants into groups facilitated data collection over a range of community views at the group, rather than the individual, level. Our study was not intended to be representative, but rather shed light on the important issues impacting people’s lives and livelihoods. The findings provide insight into the experience of living in PFAS-contaminated areas and can be used to better inform risk communication, and engagement with communities in future. This qualitative exploratory study has been used to inform the development of questions for a larger cross-sectional survey administered in affected communities.

In Australia and globally, PFAS is increasingly recognised as a long-term contaminant of land and water. Affected communities may face a spectrum of negative mental and physical effects related to uncertainty around long-term health outcomes, social and economic disruption, and a distrust of government’s ability to regulate and remediate environmental contamination. Better understanding of members’ experiences and perceptions of living in PFAS affected communities will contribute to strategies to address the mental health consequences and reduce future health costs, while improving risk communication and community engagement strategies.
